# The Role of Omega-3 Polyunsaturated Fatty Acids on Sarcopenia and Aging Muscle

**DOI:** 10.3390/ijerph23030355

**Published:** 2026-03-11

**Authors:** Behzad Varamini, Jonah O. Yang, Benjamin J. Merry, Daniel J. Dau

**Affiliations:** School of Science, Technology and Health, Biola University, La Mirada, CA 90639, USA

**Keywords:** omega-3 polyunsaturated fatty acids, sarcopenia, muscle protein synthesis, EPA, DHA, aging, resistance training, inflammation, mTOR signaling

## Abstract

**Highlights:**

**Public health relevance—How does this work relate to a public health issue?**

- Sarcopenia affects approximately 15% of community-dwelling older adults worldwide and is associated with increased risks of falls, frailty, functional decline, and mortality.
- Chronic low-grade inflammation, anabolic resistance, and mitochondrial dysfunction in aging populations create an urgent need for accessible nutritional strategies to preserve muscle health.

**Public health significance—Why is this work of significance to public health?**

- This review synthesizes mechanistic and clinical evidence demonstrating that omega-3 PUFA supplementation, particularly at doses exceeding 2 g/day, can support muscle mass and function in older adults through anti-inflammatory, anabolic, and mitochondrial pathways.
- The findings highlight that omega-3 supplementation is most effective when combined with resistance exercise as part of a multimodal approach, informing how interventions should be designed for aging populations.

**Public health implications—What are the key implications or messages for practitioners, policy makers and/or researchers in public health?**

- Clinicians and policymakers should consider omega-3 PUFA supplementation as a safe, accessible complement to exercise-based strategies for managing sarcopenia, particularly in populations with elevated inflammatory burden.
- Future public health guidelines and research should prioritize establishing optimal dosing strategies, clarifying sex-specific responses, and evaluating omega-3 supplementation within multimodal intervention frameworks.

**Abstract:**

Sarcopenia, characterized by the progressive loss of skeletal muscle mass, strength, and function, represents a major public health challenge in aging populations. This condition affects approximately 10–16% of community-dwelling older adults and is associated with increased risks of falls, frailty, functional decline, and mortality. The pathogenesis of sarcopenia involves chronic low-grade inflammation (inflammaging), oxidative stress, mitochondrial dysfunction, and anabolic resistance. Omega-3 polyunsaturated fatty acids (PUFAs), particularly eicosapentaenoic acid (EPA) and docosahexaenoic acid (DHA), have emerged as promising nutritional interventions due to their anti-inflammatory properties and potential anabolic effects on skeletal muscle. This comprehensive review evaluates the current evidence on omega-3 PUFA supplementation for the attenuation and management of sarcopenia. Mechanistically, omega-3 PUFAs appear to enhance muscle protein synthesis through activation of the mTOR-p70S6K signaling pathway, reduce inflammation via specialized pro-resolving mediators (SPMs), improve mitochondrial bioenergetics, and attenuate muscle disuse atrophy. Clinical trials demonstrate that omega-3 supplementation, particularly at doses exceeding 2 g/day of combined EPA and DHA, can increase thigh muscle volume, handgrip strength, and one-repetition maximum strength in older adults. When combined with resistance exercise training, the benefits appear more pronounced, especially in women. However, heterogeneity in study designs, intervention durations, dosages, and outcome measures has produced some conflicting results. Large-scale trials, such as the MAPT study, have shown null findings for long-term supplementation alone, suggesting that omega-3s may be most effective as part of multimodal interventions. The evidence also supports benefits in clinical populations at risk for muscle wasting, including cancer patients experiencing cachexia and individuals with neuromuscular disorders. Future research should focus on identifying optimal dosing strategies, understanding sex-specific responses, and elucidating the mechanisms underlying the synergistic effects of omega-3s with exercise. Overall, omega-3 PUFA supplementation represents a safe, accessible, and potentially effective nutritional strategy for attenuating muscle decline in aging and clinical populations, though its benefits appear most pronounced when combined with resistance exercise as part of a multimodal approach.

## 1. Introduction

Sarcopenia is a geriatric syndrome defined by the progressive, age-related loss of skeletal muscle mass and strength. This condition causes substantial morbidity in older adults and is associated with a high risk of adverse outcomes, including functional decline, falls, frailty, and increased mortality [1]. Epidemiological estimates indicate that roughly 10–16% of community-dwelling older adults worldwide are affected by sarcopenia, with even higher prevalence in those with chronic diseases [2]. The 2019 revised European Working Group on Sarcopenia in Older People (EWGSOP2) consensus established criteria incorporating low muscle strength as the primary parameter, with confirmation through low muscle quantity or quality, highlighting the clinical significance of functional decline beyond simple muscle mass loss [3]. Given the aging of populations globally, sarcopenia represents a growing public health challenge, underscoring the need for effective strategies to preserve muscle health in the elderly [4].

The pathogenesis of sarcopenia involves complex interactions of systemic and local factors. One key mechanism is chronic low-grade inflammation that accompanies aging, sometimes termed “inflammaging,” which creates a catabolic environment for muscle tissue [5]. Elevated levels of pro-inflammatory cytokines such as tumor necrosis factor-alpha (TNF-α), interleukin-1β (IL-1β), and interleukin-6 (IL-6) in older individuals can activate proteolytic pathways, particularly the ubiquitin-proteasome system, accelerating muscle protein breakdown and inhibiting muscle regeneration [6]. The relationship between inflammation and sarcopenia has been compellingly demonstrated in recent research [7,8]. It is important to distinguish this chronic, systemic inflammation from the acute, transient inflammation that occurs following exercise-induced muscle damage, which results in temporary loss of strength and limited range of motion [9]. Unlike inflammaging, acute exercise-induced inflammation is an adaptive response that plays a critical role in muscle remodeling and hypertrophy. Indeed, resistance exercise is one of the most effective interventions for reducing chronic inflammation and attenuating sarcopenia in older adults. However, when superimposed on a background of chronic low-grade inflammation, the cumulative inflammatory burden may contribute to the progressive erosion of muscle mass and function.

On a related note, oxidative stress results when reactive oxygen species (ROS) are overproduced due to increased oxygen uptake and heightened metabolic rates within active myocytes. These ROS can attack biological macromolecules, leading to tissue damage and inflammation [10]. Studies have correlated oxidative stress with sarcopenia via age-related mitochondrial dysfunction in muscle cells [11]. Mitochondrial dysfunction contributes to sarcopenia pathogenesis through the overproduction of ROS in muscle cells, resulting in myocyte degeneration [12]. The intricate relationship between mitochondrial health and muscle protein metabolism has become increasingly recognized, with recent evidence demonstrating that mitochondria serve not only as cellular powerhouses but also as critical regulators of anabolic signaling [13].

In parallel, older adults often exhibit anabolic resistance—a blunted muscle protein synthetic response to otherwise potent anabolic stimuli like amino acids or resistance exercise [14]. In aging muscle, the uptake and utilization of dietary protein are less effective at stimulating muscle protein synthesis, partly due to impairments in anabolic signaling [15]. Inflammatory processes may further exacerbate this anabolic resistance, as increased inflammatory signaling in muscle can interfere with insulin- and amino acid-mediated anabolic pathways [16]. The combination of chronic inflammation and anabolic resistance creates a vicious cycle that promotes muscle wasting in the elderly. Breaking this cycle is critical for preventing or slowing sarcopenia. Beyond exercise—the most effective countermeasure but not feasible for all older patients—there is a recognized need for additional interventions targeting these biological mechanisms [17].

Omega-3 polyunsaturated fatty acids (PUFAs) are a class of dietary unsaturated lipids characterized by a double bond between the third and fourth carbons from the omega (methyl) end [18]. The three most predominantly studied omega-3 fats—eicosapentaenoic acid (EPA), docosapentaenoic acid (DPA), and docosahexaenoic acid (DHA)—have demonstrated numerous health benefits in humans and animals, ranging from cardiovascular health to neurodevelopment [19]. These long-chain omega-3s, abundant in fatty fish and fish oil supplements, give rise to lipid mediators that are less inflammatory, or even anti-inflammatory, compared to those derived from omega-6 fatty acids [20].

Omega-3 PUFAs have emerged as a promising nutritional strategy to mitigate sarcopenia, due in large part to their anti-inflammatory and muscle anabolic properties. EPA and DHA competitively inhibit the conversion of arachidonic acid (an omega-6 PUFA) into pro-inflammatory eicosanoids, thereby reducing the production of muscle-damaging inflammatory cytokines [21]. Notably, omega-3 fatty acids also modulate cell signaling in muscle, potentially improving muscle mass and strength by mediating anabolic signaling and reducing inflammation-induced oxidative damage in muscle fibers [22]. Furthermore, omega-3 PUFAs produce specific metabolites with potent anti-inflammatory properties. Specialized pro-resolving mediators (SPMs), including resolvins, maresins, and protectins, represent a class of omega-3-derived anti-inflammatory agents that actively promote the resolution of inflammation rather than simply suppressing it [23]. Studies have also identified pathways by which omega-3 PUFAs modulate the production of lipoxygenase and cytochrome P450-derived lipid metabolites known for their anti-inflammatory properties [24,25].

These promising mechanisms have led to increased research focused on understanding omega-3 PUFA and their ability to counteract age-related skeletal muscle mass depletion [26,27]. The purpose of this comprehensive review is to survey the effects of omega-3 PUFA supplementation on sarcopenia, examining evidence across multiple outcomes including muscle mass, strength, physical function, and inflammatory biomarkers. This paper summarizes the most recent literature pertaining to the impact of omega-3 PUFA supplementation on muscle synthesis, exercise-induced inflammation, and mitochondrial function, emphasizing the interactions between omega-3 PUFAs and the prevention of sarcopenia in aging populations.

## 2. Molecular Mechanisms of Omega-3 Action on Skeletal Muscle

### 2.1. Enhancement of Muscle Protein Synthesis via mTOR Signaling

The primary means by which omega-3 fatty acids positively impact skeletal muscle mass is through incorporation of EPA and DHA into membrane phospholipids of the sarcolemma and intracellular organelles [28]. Enrichment of EPA and DHA in these membrane phospholipids is linked to enhanced rates of muscle protein synthesis, decreased expression of factors that regulate muscle protein breakdown, and improved mitochondrial respiration kinetics [29]. Smith and colleagues conducted seminal studies demonstrating that 8 weeks of omega-3 fatty acid supplementation (1.86 g/day EPA and 1.50 g/day DHA) increases rates of mixed skeletal muscle protein synthesis (MPS) in the fasted state and in response to hyperaminoacidemic-hyperinsulinemic conditions in older adults [15]. The exact mechanisms by which omega-3 fatty acids enhance MPS are not entirely clear, but appear to be mediated at least partially via increased activation of the mTOR-p70S6K signaling pathway [30]. While several plausible mechanisms have been proposed, including mTORC1 translocation, GPR120-mediated signaling, and NF-κB retention, the evidence supporting these pathways remains largely observational, and the precise molecular basis for omega-3-induced anabolism has yet to be conclusively established through direct mechanistic studies.

Similarly, the mechanistic target of rapamycin complex 1 (mTORC1) is considered an integral control point for muscle cell growth [31]. McGlory and colleagues have proposed several potential mechanisms underlying omega-3 effects on skeletal muscle, including: (1) translocation of mTORC1 with the lysosome to the membrane in close proximity to amino acid transporters; (2) enhanced adenosine diphosphate (ADP) sensitivity and altered reactive oxygen species emissions; (3) G-protein coupled receptor 120 (GPR120)-mediated signaling; (4) cytosolic retention of nuclear factor kappa B (NF-κB) preventing upregulation of proteolytic pathways; and (5) altered lipid raft formation that serves as signaling platforms [28]. Yoshino et al. provided transcriptomic evidence that n-3 PUFA therapy results in small but coordinated changes in gene expression, with increased expression of pathways regulating mitochondrial function and decreased expression of inhibitory pathways on mTOR, thereby supporting skeletal muscle anabolism [32].

### 2.2. Anti-Inflammatory and Pro-Resolving Actions

Omega-3 PUFAs exert potent anti-inflammatory effects through multiple mechanisms [33]. EPA and DHA serve as substrates for the synthesis of specialized pro-resolving mediators (SPMs), including E-series resolvins (derived from EPA), D-series resolvins, protectins, and maresins (derived from DHA) [23]. Unlike traditional anti-inflammatory drugs that simply suppress inflammatory processes, SPMs actively promote the resolution of inflammation and tissue repair. This distinction is particularly relevant in the context of sarcopenia, where chronic unresolved inflammation (inflammaging) contributes to muscle catabolism [34].

At the molecular level, omega-3 PUFAs inhibit NF-κB activation, probably via membrane-mediated actions, and they activate peroxisome proliferator-activated receptors (PPARs), which physically interfere with NF-κB translocation to the nucleus [35]. NF-κB upregulates synthesis of genes encoding many proteins involved in the inflammatory response, including cytokines, chemokines, cyclooxygenase-2 (COX-2), and matrix metalloproteinases, as well as muscle ring finger protein (MuRF), which activates the ubiquitin-proteasome system [36]. Hence, the anti-inflammatory and inflammation-resolving actions of EPA and DHA act to promote muscle protein synthesis and decrease muscle protein breakdown.

### 2.3. Mitochondrial Effects

Recent research has highlighted the importance of mitochondrial function in muscle health and the potential for omega-3 PUFAs to modulate mitochondrial bioenergetics [37]. Lalia et al. demonstrated that n-3 PUFA supplementation (3.9 g/day for 16 weeks) in older adults significantly reduced mitochondrial oxidant emissions while increasing protein synthesis rates in mitochondrial and sarcoplasmic pools [13]. Importantly, n-3 PUFA increased post-exercise mitochondrial and myofibrillar protein synthesis in older adults, suggesting a potential mechanism for enhanced exercise adaptations.

Herbst et al. found that 12 weeks of n-3 PUFA supplementation increased mitochondrial sensitivity to ADP, a critical regulator of oxidative phosphorylation [29]. McGlory et al. showed that omega-3 supplementation attenuates reductions in mitochondrial protein content and respiration during muscle disuse, independent of changes in mitochondrial H_2_O_2_ emissions [38]. Given that cytosolic protein synthesis is an energetically costly process and mitochondria are the primary source of cellular energy, retrograde signaling mechanisms may exist to maintain the balance between cytosolic and mitochondrial protein synthesis [39]. Therefore, maintenance of mitochondrial bioenergetics with omega-3 fatty acids may be an important mechanism underlying their protective effects on muscle mass.

## 3. Omega-3 and Muscle Mass

Muscle growth is a key area of interest across many life stages and is particularly relevant in older adults dealing with sarcopenia. At every stage of adult life, maintaining muscle mass through resistance exercise provides numerous health benefits, including increased bone density, higher resting metabolic rate, improved movement control, lower blood pressure, and enhanced mental health [40]. However, as humans age, a decline in endocrine function and diminished levels of testosterone, estrogen, and growth hormones contribute to decreasing muscle mass. Sarcopenia has been associated with a decline in motor skills and functional mobility [41], as well as increases in immune suppression, cardiovascular risk, metabolic disease, and osteoporosis [42]. The loss of mobility associated with sarcopenia greatly increases morbidity and mortality [22].

Numerous studies have examined the effects of omega-3 supplementation on muscle mass (Table 1). In particular, several studies have demonstrated that omega-3 promotes skeletal muscle growth via enhanced muscle protein synthesis. In a landmark study, Smith et al. [43] conducted a 6-month randomized controlled trial in 60 healthy older adults (ages 60–85) and found that fish oil-derived n-3 PUFA therapy (1.86 g EPA + 1.50 g DHA daily) increased thigh muscle volume by 3.6% (95% CI: 0.2%, 7.0%) compared to the corn oil control group. This study provided evidence that omega-3 supplementation can produce statistically significant gains in muscle volume in sedentary older adults without concurrent exercise intervention, though functional outcomes were not directly assessed as primary endpoints.

More recently, Xu et al. [44] conducted a randomized, double-blind, placebo-controlled trial in 200 older Chinese adults, demonstrating that 6 months of fish oil supplementation (1.34 g EPA + 1.07 g DHA daily) resulted in significant increases in thigh circumference, total skeletal muscle mass, and appendicular skeletal muscle mass compared to control. These effects remained significant even after height correction, supporting the direct anabolic effects of omega-3 PUFAs on muscle tissue.

Studies have also examined omega-3 supplementation in clinical populations prone to muscle mass loss, such as cancer patients and individuals with neuromuscular disorders. Murphy et al. [49] found that 45 days of omega-3 supplementation at 2.5 g/day prevented intramuscular fat infiltration in cancer patients undergoing chemotherapy. Similarly, a study by the same group demonstrated that nutritional intervention with 2.2 g/day of EPA was effective in maintaining skeletal muscle mass in patients with non-small cell lung cancer compared to standard care [45]. In children with neuromuscular disorders, Rodríguez-Cruz et al. [46] found that 6-month supplementation with omega-3 PUFA at 2.9 g/day correlated with both higher fat mass and lean mass compared to control, along with improvements in insulin sensitivity.

The protective effects of omega-3s extend to situations of muscle disuse. McGlory et al. [38] demonstrated in a study of young women that omega-3 supplementation (5 g/day; 3 g EPA + 2 g DHA) attenuated the decline in muscle volume during 2 weeks of unilateral leg immobilization (8% decline vs. 14% in control). Notably, following 2 weeks of recovery, participants in the omega-3 group fully recovered muscle volume while the control group did not. This protection against disuse atrophy was associated with higher rates of myofibrillar protein synthesis throughout the immobilization and recovery periods.

Meta-analyses have attempted to synthesize the heterogeneous literature on omega-3 and muscle mass. Huang et al. [50] analyzed 10 randomized controlled trials and found minor but significant benefits for muscle mass gain (0.33 kg; 95% CI: 0.05, 0.62) with omega-3 supplementation. Subgroup analyses suggested that doses exceeding 2 g/day may be more effective for muscle mass gain (0.67 kg; 95% CI: 0.16, 1.18). Bird et al. [51] conducted a scoping systematic review and meta-analysis including 66 studies and found a positive effect of omega-3 supplementation on overall body muscle mass, though they noted that small study sizes and heterogeneity limit applicability of findings specifically for sarcopenia prevention.

## 4. Omega-3 and Muscle Strength

While muscle mass loss is a critical component of sarcopenia, it is distinct from the loss of muscle strength, termed dynapenia. As Clark and Manini proposed, the loss of muscle strength in older adults is only partially explained by muscle mass reductions [52]. Instead, strength decline is associated with impairments in neural activation, excitation-contraction coupling, and intrinsic muscle quality. This distinction is important, as studies show that the rate of strength loss often exceeds the rate of muscle mass loss, and interventions targeting mass alone may not fully preserve functional performance [53]. Omega-3 PUFA supplementation has been explored for its role in mitigating dynapenia by supporting neuromuscular activation, mitochondrial function, and resistance training adaptations.

Several studies have investigated whether omega-3 supplementation increases muscle strength in older adults, particularly when paired with resistance training (Table 2). Kunz et al. [54] conducted a randomized controlled trial in 63 healthy older adults (mean age 71 years) and found that 6 months of fish oil supplementation (4 g/day EPA + DHA) resulted in a 7.5% increase in knee extension 1-RM compared to 3.1% in the placebo group (*p* = 0.039). While the mechanisms behind this benefit are not fully understood, researchers proposed that omega-3 fatty acids enhance the body’s acute anabolic response to exercise and may upregulate mitochondrial energy metabolism to satisfy energetic demands during muscle contraction. Importantly, as Kunz et al. [54] observed no concurrent changes in muscle mass, these strength gains likely reflect neuromuscular or qualitative adaptations rather than reversal of sarcopenia per se, consistent with the distinction between dynapenia and sarcopenia.

The interaction between omega-3 supplementation and resistance training has been examined in several trials. Rodacki et al. [55] studied 45 elderly women (~64 years) undergoing a 90-day strength training program and found that fish oil supplementation (2 g/day EPA + DHA) led to 20% greater improvement in lower body 1-RM strength compared to training with a placebo, along with better balance and functional test performance. Strandberg et al. [56] found that a diet rich in DHA omega-3s combined with 24 weeks of lower-body resistance training led to greater type II fiber cross-sectional area and leg press power compared to training alone in recreationally active older women.

An important finding from the literature is the apparent sex difference in response to omega-3 supplementation. Da Boit et al. [57] conducted a well-designed randomized controlled trial in 102 older adults (50% female) undergoing 18 weeks of resistance training with or without fish oil supplementation (3 g/day; 1.3 g EPA + 0.3 g DHA). The study found sex-specific effects: omega-3 supplementation augmented gains in muscle function and quality in older women (approximately 18% greater isokinetic torque vs. placebo) but not in older men. This sexual dimorphism may be attributed to differential enrichment of n-3 PUFA in cell membranes after supplementation, or to women’s greater capacity for improvement given their typically lower baseline muscle function [65].

However, not all studies have demonstrated benefits of omega-3 supplementation on muscle strength. Brook et al. [58] conducted a within-subject, double-blind, placebo-controlled trial in 15 older women (65–75 years) undergoing 6 weeks of unilateral leg training and found no additional strength benefit from omega-3 supplementation (~3 g/day EPA + DHA) beyond training alone, though omega-3 tended to reduce training-induced inflammation. The large-scale MAPT (Multidomain Alzheimer Preventive Trial) study by Rolland et al. [59] examined 1680 sedentary older adults (>70 years) receiving 0.8 g DHA + 0.225 g EPA daily for 3 years and found no significant differences in handgrip or chair-stand strength between omega-3 and placebo groups, suggesting that low-dose, long-term supplementation alone may be insufficient to improve strength outcomes in this population.

Recent meta-analyses have attempted to clarify these mixed findings. Santo André et al. [66] conducted a systematic review and meta-analysis finding small but statistically significant benefits of n-3 PUFA on lower-body strength and muscle function (as measured by timed up-and-go and sit-to-stand tests). Tseng et al. [67] performed a network meta-analysis of randomized controlled trials and concluded that n-3 PUFA supplementation significantly improved timed up-and-go performance by approximately 0.30 s, though they noted considerable heterogeneity among studies. A more recent systematic review by Timraz et al. [68] found that omega-3 supplementation had no significant effect on handgrip strength in healthy older adults without exercise intervention, but may increase muscle mass, suggesting dose–response and intervention-type effects merit further investigation.

## 5. Effects on Inflammatory and Oxidative Stress Biomarkers

Omega-3 PUFAs have been linked to decreased levels of post-exercise biomarkers for inflammation and oxidative stress in muscles, a beneficial outcome that may be effectively utilized in treating sarcopenia [67]. Omega-3 fatty acids reduce the production of inflammatory signaling molecules and downregulate the transcription of inflammatory cytokines and adhesion molecules, leading to a decrease in overall inflammation within muscle tissues [9].

Gray et al. [69] investigated the effect of fish oil supplementation on exercise-induced biomarkers for oxidative stress in 20 male participants over 6 weeks. Fish oil supplementation significantly reduced blood levels of thiobarbituric acid reactive substances (TBARS) and hydrogen peroxide-induced DNA damage, both biomarkers of oxidative stress. Bloomer et al. [70] examined the effect of EPA/DHA supplementation (2.2 g EPA + 2.0 g DHA daily) in exercise-trained men and found significant reductions in inflammatory biomarkers including C-reactive protein (CRP), IL-6, and TNF-α compared to placebo after 6 weeks of supplementation.

Dalle et al. [60] examined 23 older adults with knee osteoarthritis receiving 0.95 g/day omega-3 (540 mg EPA + 410 mg DHA) for 18 weeks combined with supervised exercise. The omega-3 group demonstrated improved knee extensor isometric strength despite no difference in muscle volume compared to placebo, and IL-6 levels were significantly decreased with omega-3 supplementation. This finding suggests that the benefits of omega-3 on muscle strength may be partially mediated through anti-inflammatory mechanisms rather than purely hypertrophic effects.

Alkhedhairi et al. [61] examined the effects of krill oil supplementation (4.0 g/day providing 193 mg/g EPA and 96 mg/g DHA) on muscle function in 102 adults aged >65 years. After 6 months, the krill oil-supplemented group showed increased maximal torque, grip strength, and muscle thickness compared to placebo, along with reductions in inflammatory markers. The phospholipid form of omega-3s in krill oil may enhance bioavailability and tissue incorporation, potentially explaining the positive findings.

However, not all research has demonstrated consistent anti-inflammatory effects. Cornish et al. [5] studied 23 older men undergoing 12 weeks of whole-body resistance training with or without 3.0 g/day EPA/DHA supplementation and found no significant changes in inflammatory cytokines in the supplemented group compared to the control. These inconsistencies may relate to baseline inflammatory status, as individuals with higher inflammation at baseline may show more pronounced responses to omega-3 supplementation [71]. This observation suggests that omega-3 supplementation may be most beneficial in populations characterized by elevated inflammatory burden.

## 6. Discussion and Clinical Implications

The evidence reviewed here suggests that omega-3 PUFA supplementation has potential benefits for multiple components of sarcopenia, including muscle mass, strength, and physical function. However, the heterogeneity of findings across studies warrants careful interpretation and consideration of several key factors that may influence outcomes.

Dosing considerations represent a critical factor. Meta-analyses suggest that doses exceeding 2 g/day of combined EPA and DHA appear more effective for muscle mass outcomes [50]. The seminal studies by Smith et al. [15,43] used approximately 3.36 g/day (1.86 g EPA + 1.50 g DHA), while the negative MAPT trial used only about 1 g/day, suggesting a dose–response relationship. Furthermore, the duration of supplementation matters—given the approximately 16-week lifespan of erythrocytes, this timeframe may represent a biologically relevant minimum for achieving steady-state enrichment of omega-3s in membrane phospholipids [72].

The interaction between omega-3 supplementation and exercise training deserves particular attention. Several studies demonstrate enhanced benefits when omega-3s are combined with resistance training, particularly in women [55,57]. This synergy may reflect the ability of omega-3s to enhance the anabolic response to exercise by sensitizing muscle to amino acid availability and reducing inflammation-associated anabolic resistance. From a practical standpoint, omega-3 supplementation appears to be most beneficial as part of a multimodal approach that includes exercise rather than as a standalone intervention.

Sex-specific responses to omega-3 supplementation have emerged as an important consideration. Da Boit et al. [57] reported benefits in women but not men, and women appear to have greater capacity to synthesize EPA and DHA from precursors [65]. This sexual dimorphism may have important implications for clinical recommendations and study design.

The evidence for omega-3 benefits in clinical populations experiencing or at risk for muscle wasting—such as cancer cachexia, chronic obstructive pulmonary disease, and neuromuscular disorders—is generally supportive [45,46,49]. In these populations, the anti-inflammatory properties of omega-3s may be particularly valuable given the heightened inflammatory burden associated with disease-related catabolism. EPA appears particularly effective for muscle preservation in cancer cachexia, possibly through its inhibition of proteolysis-inducing factor and IL-6 suppression [73].

While promising evidence exists, several limitations of the current evidence base warrant consideration. First, significant heterogeneity exists across studies in terms of intervention durations, omega-3 dosages, formulations, and outcome measures, making it difficult to draw universal conclusions about the precise efficacy of omega-3 supplementation or to directly compare results across trials. Second, although evidence suggests that doses exceeding 2 g/day are more effective and that the approximately 16-week erythrocyte lifespan provides a biologically relevant minimum timeframe, optimal dosing strategies and supplementation durations have not been definitively established; notably, the ineffectiveness of low-dose supplementation (~1 g/day) in the large-scale MAPT trial raises questions about the benefit of long-term, low-dose use. Third, the observed sex-specific differences in response—with women showing more pronounced benefits than men in several trials—suggest that clinical recommendations may ultimately need to be tailored by sex, though the current evidence base remains insufficient to support such distinctions. Fourth, the anti-inflammatory benefits of omega-3 supplementation may depend on an individual’s baseline inflammatory status, with those exhibiting elevated inflammation potentially deriving greater benefit; this raises the question of whether omega-3 supplementation functions as a general preventive strategy or is more appropriately targeted toward at-risk populations. Finally, the most consistent benefits have been observed when omega-3 supplementation is combined with resistance exercise training, reinforcing the view that omega-3s may serve best as a supportive component within a multimodal intervention rather than as a standalone treatment for sarcopenia.

## 7. Conclusions

Omega-3 PUFAs have shown potential in promoting muscle growth and preventing the molecular and physiological events associated with age-related sarcopenia through multiple mechanisms. The anti-inflammatory properties of omega-3 PUFAs, their ability to promote muscle protein synthesis via mTOR pathway activation, improvement of mitochondrial function, and reduction in oxidative stress are key mechanisms underlying these benefits. While further research is needed to fully elucidate optimal dosing strategies and identify populations most likely to benefit, current evidence suggests that omega-3 supplementation, particularly at doses exceeding 2 g/day of combined EPA and DHA, could be a valuable strategy for improving muscle mass and function in older adults.

The implications of omega-3 PUFAs extend beyond muscle health to impact overall physical performance and quality of life. The ability to enhance muscle strength and endurance can contribute to improved mobility and reduced risk of falls in the elderly, thereby fostering greater independence and reducing healthcare costs associated with age-related physical decline, though it should be noted that improvements in strength do not necessarily indicate reversal of sarcopenia unless accompanied by gains in muscle mass. For athletes and physically active individuals, omega-3 supplementation may facilitate faster recovery times and reduced muscle soreness, making it a valuable addition to their dietary regimen.

In light of the growing body of evidence supporting the benefits of omega-3 PUFAs, integrating these fatty acids into public health recommendations and dietary guidelines for older adults could be beneficial. Considering their natural occurrence in foods such as fatty fish, flaxseeds, and walnuts, as well as their availability in supplement form, omega-3 PUFAs are accessible and can be incorporated into a variety of dietary patterns.

Future research should aim to determine optimal dosages and forms of omega-3 supplementation for different populations, explore the potential benefits of earlier intervention throughout the lifespan, and investigate synergistic effects with other nutrients and exercise interventions. Additionally, studies should adopt more uniform designs to facilitate direct comparisons and employ longer follow-up periods to assess sustained benefits. Understanding sex-specific responses and identifying biomarkers that predict individual responsiveness would also advance the field. Overall, the promising role of omega-3 PUFAs in muscle health underscores the importance of these nutrients in supporting healthy aging across the lifespan.

## Figures and Tables

**Table 1:** Selected Studies Examining the Effects of Omega-3 Fatty Acid Supplementation on Muscle Mass or Muscle Anabolic Outcomes.

Author (Year)	Population	Location	Omega-3 Intervention	Main Findings	Ref
Lalia et al. (2017)	12 young + older adults (65–85 years)	USA	3.9 g/day n-3 PUFA for 16 weeks	↓ Mitochondrial oxidant emissions; ↑ post-absorptive muscle protein synthesis; enhanced exercise response	[13]
Smith et al. (2011)	16 healthy older adults (>65 years, mixed sex)	USA	1.86 g EPA + 1.50 g DHA/day for 8 weeks	↑ Muscle protein synthesis rate during hyperaminoacidemic-hyperinsulinemic clamp; enhanced mTOR-p70S6K signaling	[15]
Yoshino et al. (2016)	Healthy adults >65 years	USA	1.86 g EPA + 1.50 g DHA/day for 6 months	Upregulation of mitochondrial function genes; decreased mTOR inhibitory pathway expression	[32]
McGlory et al. (2019)	20 healthy young women (22 ± 3 years)	Canada	5 g/day (3 g EPA + 2 g DHA) for 4 weeks pre-immobilization	↓ Muscle volume loss during immobilization (8% vs. 14%); ↑ myofibrillar protein synthesis; full recovery in omega-3 group	[38]
Smith et al. (2015)	60 healthy older adults (60–85 years, mixed sex)	USA	1.86 g EPA + 1.50 g DHA/day for 6 months	↑ Thigh muscle volume (+3.6%); ↑ handgrip strength (+2.3 kg); ↑ 1-RM strength (+4.0%)	[43]
Xu et al. (2022)	200 older Chinese adults (>60 years)	China	1.34 g EPA + 1.07 g DHA/day for 6 months	↑ Thigh circumference; ↑ total and appendicular skeletal muscle mass; improved TUG	[44]
Murphy et al. (2011)	Lung cancer patients undergoing chemotherapy	Canada	2.2 g/day EPA for ~10 weeks	Maintained skeletal muscle mass during chemotherapy vs. standard care	[45]
Rodríguez-Cruz et al. (2019)	Children with Duchenne muscular dystrophy	Mexico	2.9 g/day omega-3 for 6 months	↑ Lean mass; ↑ fat mass; improved insulin sensitivity	[46]
Murphy et al. (2021)	107 older adults (≥65 years) at risk for sarcopenia	Ireland	0.8 g EPA + 1.1 g DHA + leucine-enriched protein for 24 weeks	No significant effect on appendicular lean mass or MyoPS vs. leucine-protein alone	[47]
Rondanelli et al. (2022)	60 sarcopenic elderly (79.7 ± 4.8 years)	Italy	500 mg omega-3 + 2.5 g leucine + probiotic for 2 months	↑ Appendicular lean mass; improved muscle performance with combination supplement	[48]

Note: ↑ indicates increased; ↓ indicates decreased.

**Table 2:** Selected Studies Examining the Effects of Omega-3 Fatty Acids on Muscle Strength and Functional Performance in Older Adults.

Author (Year)	Population	Location	Omega-3 Intervention	Main Findings	Ref
Kunz et al. (2022)	63 healthy older adults (mean 71 years, ~50% female)	USA	4 g/day fish oil (EPA + DHA) for 6 months	↑ Knee extension 1-RM (+7.5% vs. +3.1% placebo, *p* = 0.039); no change in muscle mass	[54]
Rodacki et al. (2012)	45 elderly women (~64 years) in strength training	Brazil	2 g/day EPA + DHA for 90 days + resistance training	↑ Lower body 1-RM (+20% greater than training alone); improved balance and functional tests	[55]
Strandberg et al. (2019)	24 recreationally active older women (66 ± 1 years)	Finland/Sweden	n-3 PUFA-rich diet + 24 weeks resistance training	↑ Type II fiber cross-sectional area; ↑ leg press power vs. training alone	[56]
Da Boit et al. (2017)	102 older adults (68 ± 1 years, 50% female) + resistance training	UK	3 g/day (1.3 g EPA + 0.3 g DHA) for 18 weeks	Sex-specific effects: ↑ isometric torque (+18%) and muscle quality in women only; no effect in men	[57]
Brook et al. (2021)	15 older women (65–75 years) + unilateral leg training	UK	~3 g/day EPA + DHA for 6 weeks	No added strength benefit beyond training alone; ↓ training-induced inflammation	[58]
Rolland et al. (2019)—MAPT	1680 sedentary older adults (>70 years)	France	0.8 g DHA + 0.225 g EPA/day for 3 years	No significant effect on handgrip or chair-stand strength; no interaction with lifestyle intervention	[59]
Dalle et al. (2021)	23 older adults (66 years, 52% female) with knee OA + exercise	Belgium/France	0.95 g/day (540 mg EPA + 410 mg DHA) for 18 weeks	↑ Knee extensor isometric strength; ↓ IL-6; no change in muscle volume	[60]
Alkhedhairi et al. (2022)	102 adults >65 years	UK	4 g/day krill oil (193 mg/g EPA, 96 mg/g DHA) for 6 months	↑ Maximal torque; ↑ grip strength; ↑ muscle thickness vs. placebo	[61]
Hutchins-Wiese et al. (2013)	Postmenopausal women	USA	1.2 g EPA + DHA/day for 6 months	↑ Walking speed (+0.03–0.05 m/s); clinically relevant improvement in gait	[62]
Lee et al. (2022)	Community-dwelling older adults + resistance training	USA	Fish oil + 8 weeks resistance training	↑ Grip strength; improved physical function and blood pressure; ↓ inflammation	[63]
Alves et al. (2022)	34 sarcopenic women (≥65 years) + resistance training	Brazil	4 g/day fish oil for 14 weeks + training	↑ 6 min walk distance; ↑ peak torque (+32.5% vs. +9% placebo); ↑ muscle quality	[64]

Note: ↑ indicates increased; ↓ indicates decreased.

## Data Availability

No new data were created or analyzed in this study.
